# Ty3 reverse transcriptase complexed with an RNA/DNA hybrid shows structural and functional asymmetry

**DOI:** 10.1186/1742-4690-10-S1-P109

**Published:** 2013-10-11

**Authors:** Marion Bona, Elzbieta Nowak, Jennifer T Miller, Brian Ogendi, Marcin Nowotny, Stuart FJ Le Grice

**Affiliations:** 1 RT Biochemistry Section, HIV Drug Resistance Program, National Cancer Institute, Frederick, MD, USA; 2 SAIC-Frederick, Frederick, MD, USA; 3 Laboratory of Protein Structure, International Institute of Molecular and Cell Biology, Warsaw, 02-109, USA

## 

Retrotransposons are mobile genetic elements that replicate through an RNA intermediate. They are divided into two groups, depending on the presence of flanking long-terminal repeat (LTR) sequences. Approximately 40% of the human genome is derived from retroelements with 8% corresponding to the LTR class. Retroviruses, such as human immunodeficiency virus (HIV-1) evolved from LTR elements through acquiring the envelope genes which allow them to leave the cell and infect other cells. Reverse transcriptase (RT) is a critical enzyme of retroelements, combining DNA polymerase and RNase H activities to convert the (+) strand RNA genome into double-stranded DNA that is competent for integration. However, In contrast to the extensive structural characterization of retroviral RTs, detailed information on the enzymes from LTR-containing retrotransposons such as Ty3 of *Saccharomyces cerevisiae*, is lacking.

Ty3 belongs to the Gypsy family and its RT is perhaps the most extensively characterized LTR-retrotransposon enzyme with respect to its catalytic activities, as well as the nucleic acid duplexes with which it interacts. Although structural motifs ascribed to substrate recognition and catalysis are generally similar to those of vertebrate retroviral RTs, a notable difference is replacement of the highly-conserved active site His with Tyr in the RNase H domain of Ty3 RT. More significantly Ty3 RT lacks the connection, or tether, between its DNA polymerase and RNase H active sites, with the consequence that on the nucleic acid substrate they are separated by ~13 bp as opposed to the 17-18 bp observed for lentiviral and gammaretroviral enzymes. Structural similarity between the HIV-1 connection and its RNase H domain originally suggested the latter arose through domain duplication, while an alternative theory proposes the functional RNase H domain was acquired as a new, and more efficient element from a source outside the LTR retrotransposons. Structural and biochemical information on subdomain organization of Ty3 RT is therefore important in understanding the evolutionary divergence between these essential vertebrate retroviral and LTR-retrotransposon enzymes. To this end, we provide the first structure of Ty3 RT in complex with an RNA/DNA hybrid at 3.1 Å resolution. The fully-active enzyme is an asymmetric homodimer of 55 kDa subunits, and is formed only in the presence of the RNA/DNA substrate. Modeling the spatial separation between the DNA polymerase and RNase H active sites, coupled with site-directed mutagenesis, suggests that one active site of each subunit contributes enzyme activity.

